# Dr. James Mackintosh Hays

**Published:** 1897-06

**Authors:** 


### Obituary.

Dr. James Mackintosh Hays, of Greensboro, N. C., died at his home in that city, May 15, 1897, aged about 40 years. He was one of the rising physicians in North Carolina, was a native of Greenville county, and his early demise will be lamented by the entire medical profession of the old north state.

Dr. Hays was a man of much brightness of intellect (*North Carolina Medical Journal*) and graduated from the Medical College of South Carolina, at Charleston, and became a member of the State Medical Society in 1885. He was for three years secretary of the society, and in 1894 was elected a member of the State Board of Medical Examiners, which office he filled until his death. He was an active member of the society, being but seldom absent from a meeting and presented to the society several papers of merit, to one of which The use of the microscope in the practice of medicine, was awarded the Pittman prize. Early in his professional career he reported a case of Cesarean section, performed after the death of the mother, a living child being removed.

For some years Dr. Hays practised at Oxford, his native town. He afterward practised a short time in Washington, D. C., where he was in control of Dr. Hammond's sanitarium. Later he took a special course in diseases of the eye, and after removing to Greensboro, devoted much of his time to this branch of medicine.

Dr. Hays was vice-president of the National Confederation of State Medical Examining and Licensing Boards in 1896 and a member of the executive council in 1897. He will be greatly missed in the councils of the Confederation.

